# Common uropathogens and their antibiotic susceptibility pattern among diabetic patients

**DOI:** 10.1186/s12879-018-3669-5

**Published:** 2019-01-10

**Authors:** Hiwot Ketema Woldemariam, Dereje Assefa Geleta, Kassu Desta Tulu, Negga Asamene Aber, Melese Hailu Legese, Genet Molla Fenta, Ibrahim Ali

**Affiliations:** 1 Ethiopia Public Health Institute, Addis Ababa, Ethiopia; 2 International Organization for Migration, Addis Ababa, Ethiopia; 3 Department of Medical Laboratory Sciences, College of Health sciences, Addis Ababa University, Addis Ababa, Ethiopia; 4 Department of Medical Laboratory Sciences, College of Medicine & Health Sciences, Wollo University, Dessie, Ethiopia

**Keywords:** Urinary tract infection (UTI), Diabetes mellitus (DM), Uropathogens, Antibiotic susceptibility, Multi-drug resistance

## Abstract

**Background:**

Urinary tract infection (UTIs) is a significant health problem in diabetic patients because of the multiple effects of this disease on the urinary tract and host immune system. Complicated UTIs occur most commonly in patients with abnormal genitourinary tract. Proper investigation and prompt treatment are needed to prevent morbidity and serious life threatening condition associated with UTI and diabetes co-morbidities.

**Objective:**

To determine common uropathogens and antibiotic susceptibility patterns with associated risk factors among adult diabetic patients attending at St. Paul Specialized Hospital Millennium Medical College, Addis Ababa, Ethiopia.

**Methods:**

A hospital based, cross-sectional study was conducted from April–July 2015. A total of 248 diabetic patients with asymptomatic and symptomatic UTI were investigated for common uropathogens. Clean catch mid-stream urine specimens were collected from each study subjects. Uropathogens were isolated and identified by using conventional standard techniques. Samples were cultured on Blood agar, MacConkey agar and Sabouraud Dextrose Agar. Antibiotic Susceptibility pattern was determined on Mueller-Hinton using Kirby –Bauer disc diffusion method. The collected data and the result of the laboratory were analyzed using SPSS version 20.

**Results:**

The overall prevalence of uropathogens among diabetic patients was 56/248(22.6%) of which 21/177(11.9%) and 35/71(49.3%) had asymptomatic and symptomatic UTI respectively. *E. coli* 13/56(23.2%), *Coagulase negative Staphylococci* (CONs) 7/56(12.5%), *Enterococcus* Spp.6/56 (10.7%), *Candida albicans* 10/56(17.9%) and Non-*albicans Candida* Spp. 9/56(16.1%) were the commonest isolated uropathogens. In this study uropathogens were significantly associated with being type II diabetes patient and having previous UTI history. Both gram positive and gram negative bacteria showed resistance to most tested antibiotics. Drug resistance to two or more drugs was observed in 81.1% of bacterial isolates.

**Conclusion:**

High prevalence of uropathogens and increased rate of Multi-drug resistance was shown in this study. Therefore, continued surveillance on uropathogens prevalence and resistance rates is needed to ensure appropriate recommendations for the empirical treatment, develop rational prescription programs and make policy decisions.

## Background

Diabetes mellitus (DM) is mainly associated with urinary tract infection (UTI) especially upper-UTI [1]. This is for the reason that diabetes changes the normal host system that could be possible causes to develop UTI [2]. These include increased adherence of the microorganisms to the uroepithelial cells and granulocyte dysfunction, possibly a result of an abnormal intracellular calcium metabolism [3, 4].

Few studies have shown that the reason could be due to dysfunctional bladders contracting poorly may create static pools of urine that serve as a favorable media for bacterial growth. Other studies suggest that hyperglycemic urine (sugar in the urine) encourage to increased bacterial population and colonization in the urinary tract [5]. These and others reasons make the genitourinary system where UTI can be a cause of severe life threatening complications such as renal papillary necrosis, emphysematous cystitis, and emphysematous pyelonephritis which are common in diabetes patient that leads to renal failure in this patients [6]. The association of UTI in diabetic females is more common than males because of their anatomical structure such as shorter urethra, the absence of prostatic secretion, and perineal contamination of the urinary tract with fecal flora [7].

Fungal infections are common among patients with predisposing diseases and structural abnormalities of the urinary system which are unusual causes of UTIs in healthy individuals [8]. Although bacterial pathogens are a main cause for the majority of UTI incident in diabetic patients, demonstration of Candida spp. in urine cause a diagnostic challenge [9].

Current treatment guidelines do not distinguish treatment recommendation experience for patients with DM and those without DM. Even if it is known that patients with DM dis proportionately suffer from more frequent and severe UTIs [10, 11]. Moreover, the inappropriate use of antibiotics often results in the increased resistance of urine pathogens to most commonly used antimicrobial drugs [12].

The etiologic agents as well as the resistance rates to the most commonly prescribed drugs used in the treatment of UTIs may have also be changed over time [6]. As a result, continuous and periodic surveillance of local prevalence of uropathogens and their susceptibly pattern will have public health significance to promote proper use of the existing antibiotic drugs.

Therefore, the aim of this study was to determine the prevalence of common uropathogens and their antibiotic susceptibility pattern for bacterial isolates in diabetic patients with associated risk factors at St. Paul Specialized Hospital Millennium Medical College.

## Methods

A cross sectional study was conducted from April to July 2015 at St. Paul’s Hospital Millennium Medical Collage. The hospital is found in Addis Ababa the capital city of Ethiopia. St Paul Hospital is one of the tertiary referral and teaching hospital for the Millennium Medical College and the hospital provides services to an annual average of 200,000 people who are referred from all corners of the country. A total of 248 diabetic patients attending for diabetic follow up at diabetic clinic of St. Paul’s Hospital Millennium Medical Collage were included in the study. Diabetic patients above 18 years of age were included in the study regardless of the presence or absence of UTI symptoms. Diabetic patients who took any antibiotics for the last 14 days during data collection and patients on wheelchair, complicated psychiatric disorders and kidney transplant that cannot give specimen were excluded.

Patient specific socio demographic characteristic, main possible risk factors associated with UTIs in diabetic patients, medical histories and all other required information were collected using structured data collection sheet from the patient and the responsible physician. Blood glucose level test was carried out using COBAS Integra 400/400 chemistry analyzer machine. DM was diagnosed according to World Health Organization (WHO) 2003 [13] criteria with symptoms of diabetes plus fasting blood glucose level equal or more than 126 mg%. Therefore, the data were collected base on this standard. Symptomatic UTI were diagnosed by when the patient had one or more of the following symptoms: fever, chills, nausea, vomiting, dysuria, frequency, urgency, incontinence, and flank pain.

### Specimen collection

Freshly voided 5–10 ml of clean catch midstream urine specimens was collected using sterile, graduated, wide mouthed plastic container. All participants were well instructed on how to collect clean catch midstream urine. Also for female’s participant sterile gauze was provided to make front to back wiping to dry before urination. Specimens were kept at cold chain transportation using ice packs in a cool box after collection. All samples were analyzed immediately after arrival to laboratory to ensure that the pathogenic organisms present in the urine were isolated and to avoid over population of the pathogenic organism. Microbiological investigations of this study were done at Ethiopian Public Health Institution (EPHI) National Bacteriology and Mycology Reference Laboratory.

### Isolation and identification of Uropathogens

Urine specimens were directly inoculated onto blood agar (Oxoid, ltd) and MacConkey agar (Defico, France) using a sterile standard calibrated wire loop (0.001 ml). After 24 h aerobic incubation at 36 °C, the plates were examined macroscopically for morphological appearance as presumptive identification. A colony count of ≥10^3^CFU/ml and ≥ 10^5^ CFU/ml considered as significant bacterial count for symptomatic and asymptomatic diabetic patients respectively. Identification of bacterial isolates was carried out using colony morphology, gram reaction and biochemical test.

**Fig. 1:**
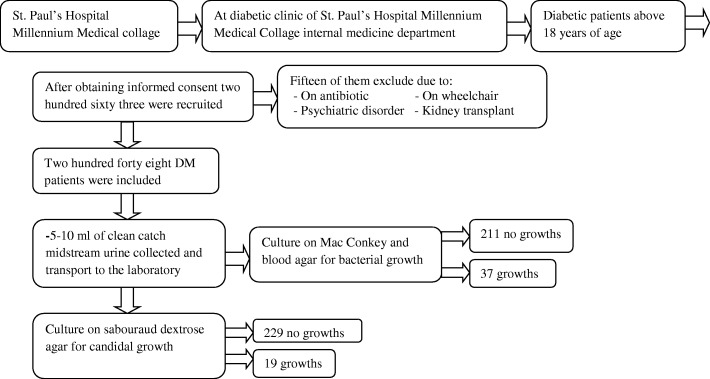
Schematic presentation of data collection and laboratory procedure

Urine samples were also inoculated onto Sabouraud dextrose agar (SDA) (Oxoid, UK) and incubated at 36 °C for 24–48 h for detection of candiduria. Significant candiduria was determined as urine culture growth ≥10^**4**^ CFU/ml. All significant candiduria were identified microscopically for morphological characteristics using germ tube production test. Data collection and laboratory procedure of this study is summarized on Fig. 1.

### Antimicrobial susceptibility testing

Antibiotic susceptibility test was carried out on each isolated bacteria using Kirby Bauer disc diffusion method according to the Clinical and Laboratory Standards Institute (CLSI: M100-S22) guidelines [14]. Bacterial suspensions were prepared by emulsifying 3–5 pure colonies in nutrient broth (Oxoid) and adjusted to 0.5 McFarland standards. A sterile cotton swab was then dipped into the suspension and swabbed on surface of Mueller-Hinton agar plate (Oxoid). Standard antibiotic discs were placed aseptically and the inoculated Mueller Hinton agar plates were incubated at 37 °C for 16–18 h. The diameters of the zones of complete inhibition were measured using mm of calipers. The isolate zone of inhibition was reported based on CLSI M100 –S22 standard as Susceptible, Intermediate and Resistant [14]. The following antibiotic discs were tested for the isolates: Ampicillin (10 μg), Amoxicillin- Clavulanic acid (10 μg), Ceftazidime (30 μg), Ceftriaxone (30 μg), Gentamicin (10 μg), Nitrofurantoin (300 μg), Cefotaxime (30 μg), Trimethoprim Sulphamethoxazole (1.25 μg), Ciprofloxacin (5 μg), Tobramycin (10 μg), Amikacin (30 μg), Penicillin (10unit) and Vancomycin (30 μg).

### Quality control

Sterility and performance of culture media were tested prior to using the culture media. Standard reference strains of *E. coli* (ATCC 25922) and *S.aureus* (ATCC 25923) were used as control for culture and sensitivity testing.

### Statistical analysis

The analysis of data obtained from this study was done using SPSS statistical software package (version 20). Percentage and frequency was used to show distribution of descriptive data using tables. Bi-variable and multi-variable analyses were done using logistic regression model for the outcome variable (significant culture positivity) and independent variables (socio demographic characteristics and health related factors)for further interpretation based on the odds ratio (OR)and level of statistical significant at *p*-value < 0.05. In addition, chi-square test was employed to see the association between current UTI status and uropathogen growth.

## Results

### Socio-demographic characteristics of the study participants

Two hundred forty eight diabetic patients were enrolled in this study. Out of248 diabetic patients 177/248(71.4%) had asymptomatic uropathogen and 71/248(28.6%) presented with symptoms of UTIs. Greater than half 136/248(54.8%) of the study subjects were females with a female to male ratio of 1.2:1. The mean age of the study subjects was 50.1 ± 14.8 years (range, 20 to 83 years). From the total study participant100/248(40.3%) of them were in the age group 56 years and above. One hundred thirty one 123/248(49.6%) participants had type I and 125/248(50.4%) had type II DM. About 90/248(36.3%) diabetic patients had at least 10 years history of diabetes. The blood glucose level of the study participant was < 126 mg/dl in99/248(39.9%) and ≥ 126 mg/dl in 149(60.1%). Majority of them were urban dwellers, 210/248(84.7%) (Table 1).

**Table 1:** Socio-demographic characteristics of diabetic patients investigated for uropathogens (*n* = 248), April to July 2015

Socio-demographic Characteristics	Classifications of variables	Frequency No. (%)
Age	20–35	51(20.6)
	36–45	45(18.1)
	46–55	52(21)
	≥56	100(40.3)
Sex	Male	112(45.2)
	Female	136(54.8)
Address:	Urban	210(84.7)
	Rural	38(15.3)
Marital Status:	Single	31(12.5)
	Married	153(61.7)
	Widowed/Divorced	64(25.8)
Literacy level:	Illiterate	84(33.9)
	Primary School	63(25.4)
	Secondary School	58(23.4)
	Higher education	43(17.3)
Types of diabetes:	Type I	123(49.6)
	Type II	125(50.4)
Duration of diabetes(in years):	< 5	65(26.2)
	5–10	93(37.5)
	≥10	90(36.3)
Blood glucose level(mg/dl):	< 126	99(39.9)
	≥126	149(60.1)
History of previous UTI:	Yes	73(29.4)
	No	175(70.6)
History of previous antibiotic:	Yes	106(42.7)
	No	142(57.3)

### Prevalence and isolated Uropathogens

The prevalence of uropathogenic microorganism infection among DM patients was 56/248(22.6%). The prevalence of the infection was significantly higher in symptomatic UTI patients 35/71(49.3%) than in asymptomatic UTI patients 21/177(11.9%), × 2 = 40.6, df = 1, *p* < 0.001). A total percent of (22.6%) diabetic patients had significant growth in their urine samples and 37/248(14.9%) of them had bacteriuria and 19/248(7.7%) had candiduria. Eight bacterial species were isolated from positive urine cultures (Table 2). *E. coli* 13/37*(*35.1%) was found to be the predominate bacterial isolate followed by *Coagulase negative staphylococci* (CONS) 7/37(18.9%), *Enterococcus* spp. 6/37(16.2%), *S.aureus* 4/37(10.8%) and others found in small number. Out of 7.7% Candiduria isolates, 10/19(52.6%) were *Candida albicans* and 9/19(47.4%) were Non-*albicans Candida* spp. More uropathogens were seen in females than in males diabetic patients. Age groups between 20 and 30 and ≥ 56 years had high frequency of uropathogens.

**Table 2:** Distribution of uropathogens in relation to sex of asymptomatic and symptomatic UTI diabetic patients (*n* = 56), April to July 2015

Uropathogens Isolated	Species	UTI status				
		Asymptomatic		Symptomatic		
		Male	Female	Male	Female	Total
		No. (%)	No. (%)	No. (%)	No. (%)	No. (%)
Bacterial	*E. coli*	1(7.7)	3(23.1)	2(15.4)	7(53.8)	13(35.1)
	CNS	2(28.6)	1(14.2)	2(28.6)	2(28.6)	7(18.9)
	*Enterococcus* spp.	1(16.7)	1(16.7)	2(33.3)	2(33.3)	6(16.2)
	*S. aureus*	1(25)	1(25)	2(50)	0(0)	4(10.8)
	*K. pneumoniae*	0(0)	0(0)	1(50)	1(50)	2(5.4)
	*P. mirabilis*	1(50)	0(0)	1(50)	0(0)	2(5.4)
	*Enterobacter* Spp.	0(0)	0(0)	2(100)	0(0)	2(5.4)
	*Citrobacter*Spp*.*	1(100)	0(0)	0(0)	0(0)	1(2.7)
	Total	7(18.9)	6(16.2)	12(32.4)	12(32.4)	37(100)
Candida	*C. albicans*	0(0)	2(20)	2(20)	6(60)	10(52.6)
	Non-*albicans Candida* Spp.	1(11.1)	5(55.6)	0(0)	3(33.3)	9(47.4)
	Total	1(5.3)	7(36.8)	2(1.5)	9(47.4)	19(100)

### Risk factors associated with urinary tract infection

Among all considered risk factor variables, type of diabetes and history of previous UTI were significantly associated with uropathogens base on (OR = 2.44, 95% CI = 1.16–5.11), (*p* = 0.02) and (OR = 2.87, 95% CI = 1.16–7.08), (*P* = 0.02), respectively as shown in Table 3.

**Table 3:** Risk factors associated with uropathogens in diabetic patients (*n* = 56), April to July 2015

Characteristics	Significant uropathogens No. (%)	Crude- OR (95.0%CI)		Adjusted-OR (95.0% CI)	
		OR(Lower-Upper)	*P*-value	OR(Lower-Upper)	*P*-value
Age:					
20–35	18(32.1)	1		1	
36–45	10(17.8)	0.52(0.21–1.29)	0.16	0 .55(0.19–1.54)	0.25
46–55	10(17.8)	0.44(0.18–1.07)	0.07	0.29(0.1–0.87)	0.03
≥ 56	18(32.1)	0.4(0.19–0.87)	0.02	0.19(0.08–0.52)	0.00
Sex:					
Male	22(39.3)	1		1	
Female	34(60.7)	1.36(0.74–2.5)	0.32	1.12(0.56–2.24)	0.75
Types of diabetes:					
Type I	22(39.3)	1		1	
Type II	34(60.7)	1.72(0.94–3.15)	0.08	2.44(1.16–5.11)	0.02
Duration of diabetes (years):					
< 5	14(25)	1		1	
5–10	20(35.7)	0.99(0.46–2.16)	0.99	1.66(0.69–4.04)	0.26
≥ 10	22(39.3)	1.18(0.55–2.53)	0.67	2.06(0.81–5.28)	0.13
Blood glucose level:					
< 126 mg/dl	21(37.5)	1		1	
≥ 126 mg/dl	35(62.5)	1.14(0.62–2.11)	0.67	0.87(0.87–1.75)	0.69
History of previous UTI:					
Yes	32(57.1)	4.91(2.61–9.24)	0.00	2.87(1.16–7.08)	0.02
No	24(42.9)	1		1	
History of previous antibiotic					
Yes	40(71.4)	1		1	
No	16(28.6)	0.21(0.11–0.4)	0.00	0.39(0.16–0.96)	0.04

*OR* Odds ratio *CI* Confidence interval

### Antibiotic susceptibility pattern of bacterial isolates

Most gram-negative bacterial isolates were observed to be sensitive to Amikacin, Tobramycin, Nitrofurantoin, Gentamicin, Ciprofloxacin, Cefotaxime, Ceftazidime, and Ceftriaxone. High bacterial resistance was observed with Ampicillin and moderate resistance to Amoxicillin-clavulanic acid. Among gram positive organisms *S. aureus* exhibited 4/4(100%) resistance to Ampicillin and penicillin, but it sowed sensitive to Nitrofurantoin and Ciprofloxacin in 100% of the isolates. *Coagulase negative Staphylococcus* (CONS) showed high level sensitivity to Amoxicillin-clavulanic acid, Cefotaxime, Ceftazidime and Nitrofurantoin 6/7 (85.7%). Ciprofloxacin 7/7 (100%), Ampicillin 5/6(83.3%) and vancomycin (83.3%) were the choice of antibiotics with greater potency for *Enterococcus* spp. However, 2/6(33.3%) of the *Enterococcus*spp. was resistance for Nitrofurantoin as shown in Tables 4 and 5.

**Table 4:** Antibiotic susceptibility pattern of gram-negative bacteria isolated from urine culture of diabetic patients (*n* = 20), April to July 2015

Bacterial Isolates	Patterns	APM	AMC	SXT	GEN	CPR	CTX	CPZ	CRO	NIT	TOB	AMK
		No.	No.	No.	No.	No.	No.	No.	No.	No.	No.	No.
*E.coli* (*n* = 13*)*	S	1(7.7)	5(38.5)	10(76.9)	10(76.9)	10(76.9)	11(84.6)	11(84.6)	11(84.6)	13(100)	12(92.3)	12(92.3)
	R	12(92.3)	8(61.5)	3(23.1)	3(23.1)	3(23.1)	2(15.4)	2(15.4)	2(15.4)	0(0)	1(7.7)	1(7.7)
*K.pneumoniae* (*n* = 2)	S	0(0)	1(50)	1(50)	2(100)	1(50)	2(100)	1(50)	2(100)	1(50)	2(100)	2(100)
	R	2(100)	1(50)	1(50)	0(0)	1(50)	0(0)	1(50)	0(0)	1(50)	0(0)	0(0)
*P.mirabilis* (*n* = 2)	S	1(50)	1(50)	1(50)	1(50)	2(100)	2(100)	2(100)	2(100)	2(100)	2(100)	2(100)
	R	1(50)	1(50)	1(50)	1(50)	0(0)	0(0)	0(0)	0(0)	0(0)	0(0)	0(0)
*Enterobacter* spp. (*n* = 2)	S	0(0)	1(50)	1(50)	1(50)	2(100)	2(100)	2(100)	2(100)	2(100)	2(100)	2(100)
	R	2(100)	1(50)	1(50)	1(50)	0(0)	0(0)	0(0)	0(0)	0(0)	0(0)	0(0)
*Citrobacter*spp. (*n* = 1)	S	1(100)	1(100)	1(100)	1(100)	1(100)	1(100)	1(100)	1(100)	1(100)	1(100)	1(100)
	R	0(0)	0(0)	0(0)	0(0)	0(0)	0(0)	0(0)	0(0)	0(0)	0(0)	0(0)
Total (*n* = 20)	S	3(15)	9(45)	15(75)	15(75)	16(80)	18(90)	17(85)	18(90)	19(95)	19(95)	19(95)
	R	17(85)	11(55)	5(25)	5(25)	4(20)	2(10)	3(15)	2(10)	1(5)	1(5)	1(5)

Abbreviations: *R* Resistance, *S* Sensitive, *AMP* Ampicillin, *AMC* Amoxicillin-clavulanic acid, *SXT* Trimethoprim-Sulfamethoxazole, *GEN* Gentamicin, *CPR* Ciprofloxacin, *CTX* Cefotaxime, *CPZ* Ceftazidime, *CRO* Ceftriaxone, *NIT* Nitrofurantoin, *TOB* Tobramycin, *AMK* Amikacin

**Table 5:** Antibiotic susceptibility pattern of gram-positive bacteria isolated from urine culture of diabetic patients (*n* = 17), April to July 2015

Bacterial Isolates	Patterns	APM	AMC	SXT	GEN	CPR	P	CTX	CPZ	NIT	VAN
		No.	No.	No.	No.	No.	No.	No.	No.	No.	No.
CONS (*n* = 7)	S	2(28.6)	6(85.7)	3(42.8)	2(28.6)	6(85.7)	2(28.6)	6(85.7)	6(85.7)	6(85.7)	NA
	I	0(0)	1(14.3)	1(14.3)	1(14.3)	1(14.3)	0(0)	1(14.3)	1(14.3)	0(0)	NA
	R	5(71.4)	0(0)	3(42.8)	4(57.1)	0(0)	5(71.4)	0(0)	0(0)	1(14.3)	NA
*Enterococcus* spp. (*n* = 6)	S	5(83.3)	NA	NA	NA	6(100)	NA	NA	NA	4(66.7)	5(83.3)
	I	0(0)	NA	NA	NA	0(0)	NA	NA	NA	0(0)	0(0)
	R	1(16.7)	NA	NA	NA	0(0)	NA	NA	NA	2(33.3)	1(16.7)
*Staphylococcus aureus* (*n* = 4)	S	0(0)	3(75)	3(75)	3(75)	4(100)	0(0)	3(75)	3(75)	4(100)	NA
	I	0(0)	0(0)	0(0)	0(0)	0(0)	0(0)	0(0)	0(0)	0(0)	NA
	R	4(100)	1(25)	1(25)	1(25)	0(0)	4(100)	1(25)	1(25)	0(0)	NA
Total (*n* = 17)	S	7(41.2)	9(81.8)	5(45.5)	5(45.5)	15(88.2)	2(18.8)	9(81.8)	9(81.8)	14(82.1)	5(83.3)
	I	0(0)	1(9.1)	1(9.1)	1(9.1)	1(5.9)	0(0)	1(9.1)	1(9.1)	0(0)	0(0)
	R	10(58.8)	1(9.1)	5(45.5)	5(45.5)	1(0)	9(81.8)	1(9.1)	1(9.1)	3(17.6)	1(16.7)

Abbreviations: *R* Resistance, *S* Sensitivem, *I* Intermediate, *NA* Not applicable, *AMP* Ampicillin, *AMC* Amoxicillin-clavulanic acid, *SXT* Trimethoprim-Sulfamethoxazole, *GEN* Gentamicin, *CPR* Ciprofloxacin, *P* penicillin, *CTX* Cefotaxime, *CPZ* Ceftazidime, *NIT* Nitrofurantoin, *VAN* vancomycin

Drug resistance for two or more drugs was observed in 18/20(85%) and 12/17(70.6%) of gram-negative and gram-positive bacteria respectively. Three *E. coli* (23.1%) and one *K. pneumoniae* (50%) among gram negative isolates and one *S. aureus* isolate (25%) among gram positive isolate shows resistance to five or more antimicrobials. The overall prevalence of multi drug resistance in both groups was 81.1% (Tables 6 and 7).

**Table 6:** Multi- Drug resistance pattern of gram-negative bacteria isolated from urine culture of diabetic patients, April to July 2015

Combination of antibiotics	Total	*E.coli*	*K.pneumoniae*	*P.mirabilis*	*Enterobacter spp.*	*Citrobacter*
AMP,AMC	7(14.3)	5 (11.9)	1(100)	–	1(16.7)	–
AMP,AMC, SXT	2 (4.1)	1(2.4)	–	–	1(16.7)	–
AMP,AMC, SXT,GEN	2 (4.1)	1(2.4)	–	–	1(16.7)	–
AMP,AMC, SXT,GEN,CPR,	1(2)	1(2.4)	–	–	–	–
AMP,AMC, SXT,GEN,CPR,CTX	1(2)	1(2.4)	–	–	–	–
AMP,AMC, SXT,GEN,CPR,CTX,CPZ	1(2)	1(2.4)	–	–	–	–
AMP,AMC, SXT,GEN,CPR,CTX,CPZ,CRO	1(2)	1(2.4)	–	–	–	–
AMP,AMC, SXT,GEN,CPR,CTX,CPZ,CRO,TOB	1(2)	1(2.4)	–	–	–	–
AMC, SXT	2 (4.1)	1(2.4)	–	–	1(16.7)	–
AMC, SXT,GEN	2 (4.1)	1(2.4)	–	–	1(16.7)	–
AMC, SXT,GEN,CPR	1(2)	1(2.4)	–	–	–	–
AMC, SXT,GEN,CPR,CTX	1(2)	1(2.4)	–	–	–	–
AMC, SXT,GEN,CPR,CTX,CPZ	1(2)	1(2.4)	–	–	–	–
AMC, SXT,GEN,CPR,CTX,CPZ,CRO	1(2)	1(2.4)	–	–	–	–
AMC, SXT,GEN,CPR,CTX,CPZ,CRO,TOB	1(2)	1(2.4)	–	–	–	–
SXT,GEN	4 (8.2)	3 (7.1)	–	–	1(16.7)	–
SXT,GEN,CPR	1(2)	1(2.4)	–	–	–	–
SXT,GEN,CPR,CTX	1(2)	1(2.4)	–	–	–	–
SXT,GEN,CPR,CTX,CPZ	1(2)	1(2.4)	–	–	–	–
SXT,GEN,CPR,CTX,CPZ,CRO	1(2)	1(2.4)	–	–	–	–
SXT,GEN,CPR,CTX,CPZ,CRO,TOB	1(2)	1(2.4)	–	–	–	–
GEN,CPR	1(2)	1(2.4)	–	–	–	–
GEN,CPR,CTX	1(2)	1(2.4)	–	–	–	–
GEN,CPR,CTX,CPZ	1(2)	1(2.4)	–	–	–	–
GEN,CPR,CTX,CPZ,CRO	1(2)	1(2.4)	–	–	–	–
GEN,CPR,CTX,CPZ,CRO,TOB	1(2)	1(2.4)	–	–	–	–
CPR,CTX	1(2)	1(2.4)	–	–	–	–
CPR,CTX,CPZ	1(2)	1(2.4)	–	–	–	–
CPR,CTX,CPZ,CRO	1(2)	1(2.4)	–	–	–	–
CPR,CTX,CPZ,CRO,TOB	1(2)	1(2.4)	–	–	–	–
CTX,CPZ	1(2)	1(2.4)	–	–	–	–
CTX,CPZ,CRO	1(2)	1(2.4)	–	–	–	–
CTX,CPZ,CRO,TOB	1(2)	1(2.4)	–	–	–	–
CPZ,CRO	1(2)	1(2.4)	–	–	–	–
CPZ,CRO,TOB	1(2)	1(2.4)	–	–	–	–
CRO,TOB	1(2)	1(2.4)	–	–		–
Total	49 (100)	42 (100)	1(100)	–	6(100)	–

*AMP* Ampicillin, *AMC* Amoxicillin-clavulanic acid, *SXT* Trimethoprim-sulphamethoxazole, *GEN* Gentamicin, *CPR* Ciprofloxacin, *CTX* Cefotaxime, *CPZ* Ceftazidime, *CRO* Ceftriaxone, *NIT* Nitrofurantoin, *TOB* Tobramycin, *AMK* Amikacin

**Table 7:** Multi- Drug resistance pattern of gram-positive bacteria isolated from urine culture of diabetic patients, April to July 2015

Combination of antibiotics	Total	CONS	*Enterococcus*	*S.aureus*
AMP,AMC	1(5.9)	–	–	1 (6.7)
AMP,AMC,GEN	1(5.9)	–	–	1 (6.7)
AMP,AMC,GEN,P	1(5.9)	–	–	1 (6.7)
AMC,GEN,P	1(5.9)	–	–	1 (6.7)
SXT,GEN	1(5.9)	2 (66.7)	–	1 (6.7)
SXT,GEN,P	2 (17.8)	1 (33.3)	–	1 (6.7)
GEN,CPR	1(5.9)	–	–	1 (6.7)
GEN,CPR,P	1(5.9)	–	–	1 (6.7)
GEN,CPR,P,CTX,	1(5.9)	–	–	1 (6.7)
GEN,CPR,P,CTX,CPZ	1(5.9)	–	–	1 (6.7)
CPR,P,CTX	1(5.9)	–	–	1 (6.7)
CPR,P,CTX,CPZ	1(5.9)	–	–	1 (6.7)
P,CTX	1(5.9)	–	–	1 (6.7)
P,CTX,CPZ	1(5.9)	–	–	1 (6.7)
CTX,CPZ	1(5.9)	–	–	1 (6.7)
NIT,VAN	1(5.9)	–	1(100)	–
Total	17 (100)	3 (100)	1(100)	15 (100)

*AMP* Ampicillin, *AMC* Amoxicillin-clavulanic acid, *SXT* Trimethoprim-sulphamethoxazole, *GEN* Gentamicin, *CPR* Ciprofloxacin, *P* penicillin, *CTX* Cefotaxime, *CPZ* Ceftazidime, *CRO* Ceftriaxone, *NIT* Nitrofurantoin, *VAN* vancomycin

## Discussion

In the present study, the overall prevalence of Uropathogens in diabetic patients was 22.6% with prevalence of 14.9% significant bacteriuria and 7.7% significant candiduria. The 14.9% prevalence of bacteriuria in our study was similar with what had been previously reported [15–18]. However, this finding is not in accordance with the results reported from India (32%) [19], Iraq (49.1%) [1] and Pakistan (51%) [20]. The reason of this difference in rate of bacterial UTI etiologies may be differences in methodology used, the environment, social habits of the community, the standard of personal hygiene and education level in each country.

Fungi, particularly candida yeasts are a common predisposing factor of UTI in diabetes mellitus patients [21, 22]. In the present study, significant candiduria was found in 7.7% similar findings have been reported in Gondar, Ethiopia (8.3%) [21] and Saudi Arabia (12%) [23].

In our study*E. coli* was the most predominant bacterial isolate and its prevalence was 35.1% this is in line with the findings in Gondar, Ethiopia (31.7%) [16], however, it was lower than previous study report by Hamdan et al and Osanyinpeju et al [17, 18]. The second frequently isolated bacteria were Coagulase negative staphylococci (CONS) (18.9%). Similar finding has been reported by Yismaw G, et al *in* Gondar, Ethiopia and who explained that the high isolation rate of CONs is due to contamination during specimen collection or processing and/or changes in pattern of infection in diabetic patients [16]. Furthermore, recently this type of bacteria is regarded as the important agent of hospital infections and also opportunist infections [24].

Appropriate management of ASB culture results in diabetic patients has been beneficial to prevent symptomatic UTI and further risk of serious UTI complications [25]. In our study, 7.3% asymptomatic bacteriuria was identified in diabetic patients. Other study in the same study area has also reported similar finding (10.4%) [15]. However, other studies have reported higher findings [5, 16–18].

In this study *C. albicans and* Non-*albicans Candida spp.* accounted for 52.6 and 47.4% respectively. A high proportion of significant candiduria 84.2% was recorded in women participants than men 15.8% respectively. Similar findings have also reported by Yismaw G et al in Gondar, Ethiopia [21] and Awwad K in Saudi Arabia [23].

In the present study, the prevalence of asymptomatic and symptomatic candiduria was 4.3 and 17.18% respectively. In our study the majority of infections by *Candida* species were symptomatic UTI, therefore anti-fungal treatment and follow up should be significant for the reason that diabetes mellitus is a predisposing factor for developing Candida UTIs [6].

In different previous study diabetes related risk factors have been proposed as an enhanced factor for UTI in patients with diabetes [6]. In this study among the variables as diabetes related risk factor for UTI, a significant association was observed for uropathogens and being type II diabetes patient and having previous UTI history. This is in accordance with prior study from Gondar, Ethiopia and elsewhere [16, 21].

In the present study, intermediate to low level resistance for one or more antimicrobial agents was observed for both gram-negative and gram-positive bacteria except for Ampicillin that shows high level of resistance. Comparatively similar findings have reported in previous studies [16, 18, 26]. The highest level of resistance to Ampicillin may be due to easily availability, low cost of the antibiotic and use of drugs without prescription. In this study, gram-negative bacteria were highly susceptible to Amikacin, Tobramycin and Nitrofurantoin at similar rate (95%) and Gentamicin, Ciprofloxacin, Cefotaxime, Ceftazidime, Ceftriaxone have also similar rate (85%). This low resistances rate might be due to these drugs are not easily available and/or relatively expensive compared to others in the study area. Therefore, these drugs could be considered as therapeutic options in the empirical treatment of UTIs in this study subjects.

In this study the predominant isolate *E. coli* showed exceedingly low susceptibility to Ampicillin (7.7%), followed by Amoxicillin-clavulanic acid (38%). Similar result has been observed in many other studies [27, 28]. The other gram- negative isolates *K. pneumoniae* showed 100% resistance to Ampicillin which is in line with other studies conducted in Gondar, Ethiopia [16], Nigeria [18] and Libya [26]. *Coagulase negative Staphylococcus* which is the second most commonly isolated bacteria showed intermediate to high level resistance to the commonly prescribed antibiotics this is relatively similar study from Iran [24] however, relatively different from the study results in Gondar, Ethiopia [16].

In the present study, S. *aureus* showed 100% susceptibility to Nitrofurantoin but 100% resistance to Ampicillin, Trimethoprim-sulphamethoxazole and penicillin. The same findings reported in a study from Pakistan [29]. This higher resistance pattern for these drugs is comparable to other reports from Gondar, Ethiopia [16] and Nigeria [18].

Multi drug resistance was observed in 81.1% of the isolated bacteria in this study. This is higher than the previous study findings in Addis Ababa (71.7%) [15] and Gondar (59.8%) [16]. This showed that multi drug resistance was found to be very high to the commonly used antibiotics in diabetic patients. Antibiotic resistance has been recognized as the consequence of inappropriate use of antibiotic [30]. Therefore, the reasons for this alarming event might be improper and incorrect administration of antimicrobial agents as empiric therapies.

## Conclusion

The overall prevalence of UTI was high. E. coli was the most predominant bacterial isolate. The risk of UTI increased intype II diabetes and having previous UTI history in this study. This study shows a large number of isolates resistance to Ampicillin and penicillin. Speciation and antifungal susceptibility investigation is crucial for the management UTI caused by fungal Candida pathogens. Routine urine culture may be considered, especially for the detection of asymptomatic bacteriuria cases in diabetic patients that may benefit from antibiotic therapy, considering their tendency to progress to symptomatic UTI, and to develop complications from UTI. It is advisable to enhance sensitization works against antibiotic inappropriate use to control the spread of multi drug resistant bacterial pathogens in the study area and beyond. As limitation this study was focused on microbiological approach. Therefore, detailed information regarding on the endocrinology of diabetic patients was not investigated.

## Abbreviations

ATCC: American type culture collection
CFU: Colony forming unit
CLSI: Clinical laboratory standards Institute
CONS: Coagulase negative staphylococci
DM: Diabetes mellitus
EPHI: Ethiopian PublicHealth Institute
SDA: Sabroud dextrose agar
SPSS: Statistical package for the social sciences
UTI: Urinary tract infection
